# Polydeoxyribonucleotide Attenuates Carbon Tetrachloride-Induced Acute Liver Injury Through A_2A_ Receptor-Related Anti-Inflammatory Responses

**DOI:** 10.3390/life16091498

**Published:** 2026-09-07

**Authors:** Il-Gyu Ko, Su Bee Park, Hyun Phil Shin, Jung Won Jeon, SeungHwan Lee

**Affiliations:** 1 Research Support Center, School of Medicine, Keimyung University, Daegu 42601, Republic of Korea; rhdlfrb@naver.com; 2 Department of Internal Medicine, Kyung Hee University Hospital at Gangdong, College of Medicine, Kyung Hee University, Seoul 05278, Republic of Korea; endobee90@gmail.com (S.B.P.); drshp@khu.ac.kr (H.P.S.); drglory@naver.com (J.W.J.); 3 Department of Surgery, Kyung Hee University Hospital at Gangdong, College of Medicine, Kyung Hee University, Seoul 05278, Republic of Korea

**Keywords:** acute liver injury, polydeoxyribonucleotide, adenosine A_2A_ receptor, macrophage accumulation, high mobility group box 1

## Abstract

**Background/Objectives:** Polydeoxyribonucleotide (PDRN) has anti-inflammatory and tissue-protective properties associated with activation of the adenosine A_2A_ receptor (A_2A_R). Although hepatoprotective actions of PDRN have previously been described, its potential association with high-mobility group box 1 (HMGB1)-related inflammatory responses and hepatic macrophage accumulation during acute liver injury (ALI) is not fully understood. This study examined whether PDRN affects HMGB1- and monocyte chemoattractant protein-1 (MCP-1)-associated responses together with hepatic macrophage accumulation in ALI. **Methods:** ALI was induced in ICR mice by carbon tetrachloride (CCl_4_) administration. PDRN was administered either alone or together with the selective A_2A_R antagonist 3,7-dimethyl-1-propargylxanthine (DMPX). Serum levels of aspartate aminotransferase, alanine aminotransferase, and lactate dehydrogenase were quantified. Histological alterations were evaluated using hematoxylin and eosin staining. Hepatic macrophage accumulation was assessed by F4/80 immunofluorescence staining. Enzyme-linked immunosorbent assays were used to quantify HMGB1, MCP-1, interleukin-10 (IL-10), cyclic adenosine monophosphate (cAMP), and A_2A_R. **Results:** PDRN significantly reduced serum markers of liver injury and attenuated histopathological damage following CCl_4_ administration. PDRN treatment also decreased F4/80-positive macrophage accumulation in the hepatic tissue. In addition, PDRN reduced HMGB1 and MCP-1 levels in both the serum and liver tissues while significantly increasing IL-10, cAMP, and A_2A_R levels. **Conclusions:** PDRN attenuated CCl_4_-induced ALI and was associated with reduced HMGB1 and MCP-1 levels, decreased hepatic macrophage accumulation, and increased A_2A_R/cAMP signaling. These findings suggest that modulation of macrophage-associated inflammatory responses may contribute to the hepatoprotective effects of PDRN.

## 1. Introduction

Acute liver injury (ALI) is a common pathological condition characterized by the rapid deterioration of hepatic function following exposure to various injurious stimuli, including viral infection, drug toxicity, alcohol consumption, and environmental toxins [1]. Excessive hepatocellular damage can trigger inflammatory responses that contribute to the progression of tissue injury and functional impairment [2,3]. Despite the advances in supportive management, effective therapeutic strategies for limiting hepatic inflammation and promoting tissue recovery remain limited.

Inflammation is increasingly recognized as a critical component of the pathogenesis of ALI. Among the mediators involved in this process, high mobility group box 1 (HMGB1) is an important regulator of hepatic inflammation. HMGB1 is a highly conserved nuclear protein that is released into the extracellular environment following cellular injury or necrosis and subsequently activates multiple inflammatory signaling pathways, promoting the recruitment of immune cells to sites of tissue damage [4,5].

Elevated HMGB1 expression has been reported in both experimental and clinical liver diseases, and it has been found to be closely associated with the severity of hepatic injury [6,7]. In addition to its direct pro-inflammatory effects, extracellular HMGB1 can promote monocyte chemoattractant protein-1 (MCP-1) production through the activation of downstream inflammatory signaling pathways rather than through a direct molecular interaction. MCP-1, in turn, plays a central role in the recruitment of monocytes and macrophages to injured tissues [8,9,10]. Thus, HMGB1-associated inflammatory signaling may contribute to MCP-1-mediated macrophage recruitment during tissue injury.

Increased MCP-1 expression facilitates macrophage accumulation within damaged liver tissues, thereby amplifying local inflammatory responses and contributing to the progression of hepatic injury [11,12]. Although hepatocyte injury, apoptosis, and pro-inflammatory cytokine production have been extensively investigated in ALI, the contribution of HMGB1-associated inflammatory signaling and macrophage accumulation to disease progression remains unclear. Therefore, modulation of HMGB1-associated inflammatory responses and macrophage recruitment has emerged as a potential therapeutic strategy for ALI.

Polydeoxyribonucleotide (PDRN) is a highly purified mixture of deoxyribonucleotide polymers derived from salmonid DNA [13,14]. Its pharmacological activity is primarily associated with activation of the adenosine A_2A_ receptor (A_2A_R), leading to increased intracellular cyclic adenosine monophosphate (cAMP) and regulation of inflammatory responses [13]. PDRN has been investigated for its tissue-reparative, angiogenic, and anti-inflammatory effects in various experimental models, and its therapeutic potential has also been explored in clinical settings [13,14,15]. In our previous study, PDRN attenuated ALI by reducing hepatocellular damage, apoptosis, and pro-inflammatory cytokine production, while enhancing A_2A_R expression [16,17].

Given the established role of A_2A_R activation in suppressing inflammatory mediator and chemokine production, these findings raise the possibility that PDRN may also influence HMGB1- and MCP-1-associated inflammatory responses during ALI. However, it remains unknown whether these hepatoprotective effects are accompanied by changes in HMGB1-associated inflammatory responses, MCP-1 expression, and hepatic macrophage accumulation during ALI.

Therefore, the present study was designed to investigate whether the hepatoprotective effects of PDRN are associated with changes in HMGB1 and MCP-1 expression and hepatic macrophage accumulation in a mouse model of ALI. We expected that the protective effect of PDRN against CCl_4_-induced ALI would be associated with reduced HMGB1 and MCP-1 expression and macrophage accumulation through A_2A_R-related signaling. To further clarify the involvement of A_2A_R activation, the selective A_2A_R antagonist 3,7-dimethyl-1-propargylxanthine (DMPX) was used.

## 2. Materials and Methods

### 2.1. Animals and Experimental Groups

Six-week-old male ICR mice (26 ± 1 g) were purchased from Orient Bio Inc. (Seongnam, Republic of Korea). To limit variability related to sex-associated hormonal fluctuations in this chemically induced ALI model, only male mice were used. During the study, mice were maintained at 23 ± 2 °C under a 12-h light/dark cycle and provided standard chow and water ad libitum. The experimental protocol was approved by the Institutional Animal Care and Use Committee of Kyung Hee University (KHUASP[SE]-20-495), and all animal procedures complied with the institutional guidelines for laboratory animal care and use. Before group assignment, the 40 mice were weighed and randomly distributed into four groups (*n* = 10 each) while maintaining comparable initial body weights among the groups. The experimental groups were designated as follows: (1) control (CON), (2) CCl_4_, (3) CCl_4_-PDRN, and (4) CCl_4_-PDRN + DMPX. Treatment was administered with group identity known to the investigator, while outcome evaluation and data quantification were performed by investigators blinded to group allocation.

### 2.2. Induction of CCl4-Induced ALI and Drug Administration

**Figure 1:**
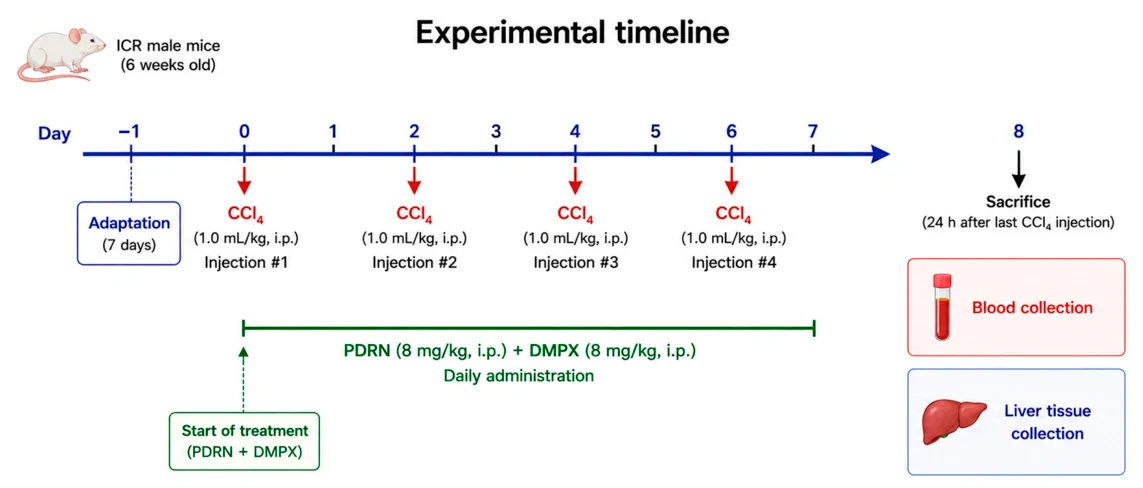
Experimental timeline.

The CCl_4_-induced ALI protocol was adapted from previously described procedures [17,18]. CCl_4_ (10 mL/kg; a preparation containing 0.4 mL CCl_4_ per 100 mL of corn oil) was injected intraperitoneally on four occasions separated by 2-day intervals, while control mice received the corresponding volume of vehicle. Beginning on the day after the first CCl_4_ challenge, PDRN (Rejuvenex^®^, PharmaResearch Products Co., Ltd., Seongnam, Republic of Korea; 8 mg/kg) was given intraperitoneally once per day for seven consecutive days. Mice assigned to the CCl_4_-PDRN + DMPX group additionally received DMPX (8 mg/kg; Sigma-Aldrich, St. Louis, MO, USA) once daily over the corresponding treatment period. All drug administrations and sample collections were performed at approximately 10:00 a.m. to minimize potential effects of circadian variation. At 24 h after the last treatment, the animals were anesthetized and euthanized, after which blood and liver specimens were obtained for biochemical, histological, immunofluorescence, and ELISA analyses. Figure 1 summarized the experimental schedule.

### 2.3. Serum Biochemical Analysis

At the experimental endpoint, blood collected from the abdominal vein was centrifuged at 3000× *g* for 15 min to obtain serum, which was maintained at −70 °C until analysis. Serum activities of aspartate aminotransferase (AST), alanine aminotransferase (ALT), and lactate dehydrogenase (LDH) were subsequently determined with an AU400 automated clinical chemistry analyzer (Olympus, Tokyo, Japan).

### 2.4. Histological Examination and Liver Injury Scoring

For histopathological assessment, liver specimens were preserved in 10% neutral-buffered formalin, processed for paraffin embedding, cut into 4-μm sections, and subjected to hematoxylin and eosin (H&E) staining. Microscopic evaluation was conducted by investigators blinded to group allocation. Hepatic injury was evaluated using a modified version of a previously reported scoring method based on the distribution and severity of parenchymal necrosis [19]. Injury was graded on a five-point scale (0–4): score 0, normal hepatic histology; score 1, hepatocyte degeneration with occasional necrotic foci; score 2, mild centrilobular necrosis surrounding the central vein and involving a portion of Rappaport zone III; score 3, established necrosis confined to zone III; and score 4, extensive confluent centrilobular necrosis extending through Rappaport zones III and II. At least four sections from each animal were examined, with four non-overlapping fields assessed per section at ×150 magnification.

### 2.5. Immunofluorescence Staining of F4/80

F4/80 immunofluorescence was carried out with modifications to previously reported procedures [20,21]. Paraffin sections of liver tissue were first cleared with xylene and then rehydrated through sequentially graded ethanol solutions. Following antigen retrieval in 10 mM sodium citrate buffer, nonspecific binding was blocked with 5% normal goat serum supplemented with 0.3% Triton X-100 (The Dow Chemical Company, Midland, MI, USA). Sections were then exposed overnight at 4 °C to rat anti-mouse F4/80 antibody (clone CI, MCA497; Bio-Rad Laboratories, Hercules, CA, USA). After incubation with the corresponding fluorescence-labeled secondary antibody, nuclei were labeled with 4′,6-diamidino-2-phenylindole (DAPI; Vector Laboratories, Burlingame, CA, USA), and the preparations were mounted using Vectashield^®^ mounting medium (Vector Laboratories, Burlingame, CA, USA).

Images were obtained with a Leica DMi8 fluorescence microscope (Leica Microsystems, Nussloch, Germany). Hepatic macrophage accumulation was quantified as the F4/80-positive area using Leica Application Suite X (LAS X), with the same image-acquisition settings applied to all groups.

### 2.6. Enzyme-Linked Immunosorbent Assay

ELISA measurements were performed using serum and liver tissue samples. For tissue-based assays, liver specimens were disrupted in chilled homogenization buffer and centrifuged to obtain the supernatant. HMGB1 and MCP-1 concentrations were assessed in both serum and liver homogenates, while IL-10, cyclic adenosine monophosphate (cAMP), and A_2A_R were evaluated in liver homogenates only. Values obtained from liver homogenates were adjusted to the total protein content of the corresponding sample. Commercial ELISA kits were used to determine HMGB1 and MCP-1 (Abbexa, Cambridge, UK), IL-10 (Abcam, Cambridge, UK), cAMP (Cayman Chemical, Ann Arbor, MI, USA), and A_2A_R (LifeSpan Biosciences, Seattle, WA, USA). Assays were conducted following the instructions supplied with each kit. Absorbance was recorded with a Multiskan GO microplate reader (Thermo Fisher Scientific, Waltham, MA, USA), and concentrations were derived from standard curves prepared using known standards.

### 2.7. Western Blot Analysis

Liver tissues were lysed in chilled RIPA buffer (Cell Signaling Technology, Danvers, MA, USA) containing 1 mM phenylmethylsulfonyl fluoride (Sigma-Aldrich, St. Louis, MO, USA). Protein aliquots of 30 μg were resolved by 12% SDS-PAGE and transferred onto nitrocellulose membranes. The membranes were blocked with 5% skim milk in Tris-buffered saline containing 0.1% Tween-20 for 1 h at room temperature. After blocking, the membranes were incubated overnight at 4 °C with rabbit anti-A_2A_R antibody (Cat. No. 53509; Cell Signaling Technology; 1:1000) and mouse anti-β-actin antibody (Cat. No. sc-47778; Santa Cruz Biotechnology, Dallas, TX, USA; 1:1000). After washing, the membranes were incubated for 1 h at room temperature with horseradish peroxidase-conjugated goat anti-rabbit IgG (Cat. No. PI-1000; Vector Laboratories, Newark, CA, USA; 1:2000) or horse anti-mouse IgG (Cat. No. PI-2000; Vector Laboratories; 1:2000), as appropriate. Immunoreactive signals were detected with an enhanced chemiluminescence detection kit (Bio-Rad Laboratories) and visualized by exposure to X-ray film followed by film development. Band intensities were quantified with Image-Pro Plus software (version 9.0; Media Cybernetics Inc., Rockville, MD, USA). Protein expression values were normalized against β-actin, and the control group was assigned a value of 1.00.

### 2.8. Statistical Analysis

Data are expressed as the mean ± standard error of the mean (SEM). Statistical analyses were conducted with SPSS version 23.0 (IBM Corp., Armonk, NY, USA). The normality of data distribution and homogeneity of variance were assessed using the Shapiro–Wilk test and Levene’s test, respectively. Differences among groups were analyzed by one-way analysis of variance (ANOVA), with subsequent comparisons performed using Tukey’s honestly significant difference (HSD) test for post hoc comparisons. A value of *p* < 0.05 was considered statistically significant. The number of biological samples used for each analysis is specified in the corresponding figure legends. All measurements were repeated at least three times to confirm reproducibility.

## 3. Results

### 3.1. PDRN Treatment Attenuated CCl_4_-Induced Hepatocellular Injury

**Figure 2:**
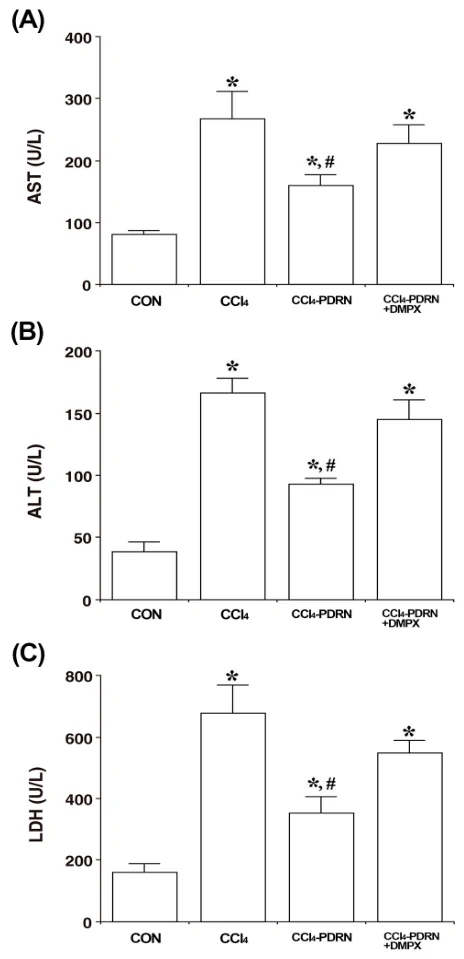
Effects of PDRN on the serum biochemical markers in CCl_4_-induced ALI (**A**–**C**) Serum AST, ALT, and LDH activities, respectively. PDRN lowered the CCl_4_-associated increases in these enzymes, whereas DMPX co-treatment attenuated this effect. Groups were designated as CON (control), CCl_4_ (CCl_4_-injected), CCl_4_-PDRN (CCl_4_ plus PDRN), and CCl_4_-PDRN + DMPX (CCl_4_ plus PDRN and DMPX). Values are expressed as mean ± SEM (*n* = 10/group). Group comparisons were performed by one-way ANOVA with Tukey’s HSD post hoc test. * *p* < 0.05 versus the control group; # *p* < 0.05 versus the CCl_4_-injected group.

To evaluate the hepatoprotective effect of PDRN, the serum AST, ALT, and LDH activities were determined after CCl_4_-induced liver injury (Figure 2). CCl_4_ administration significantly increased all three serum enzyme activities relative to the control group (*p* < 0.05), indicating substantial hepatocellular damage. Treatment with PDRN lowered these elevations relative to CCl_4_ alone (*p* < 0.05). However, DMPX co-treatment attenuated this reduction, resulting in significantly higher AST, ALT, and LDH levels than those observed in the CCl_4_-PDRN group (*p* < 0.05). Overall, these results indicate that PDRN attenuated CCl_4_-induced hepatocellular injury and that this response involved A_2A_R-related signaling.

### 3.2. PDRN Treatment Improved Histopathological Alterations in the Liver

**Figure 3:**
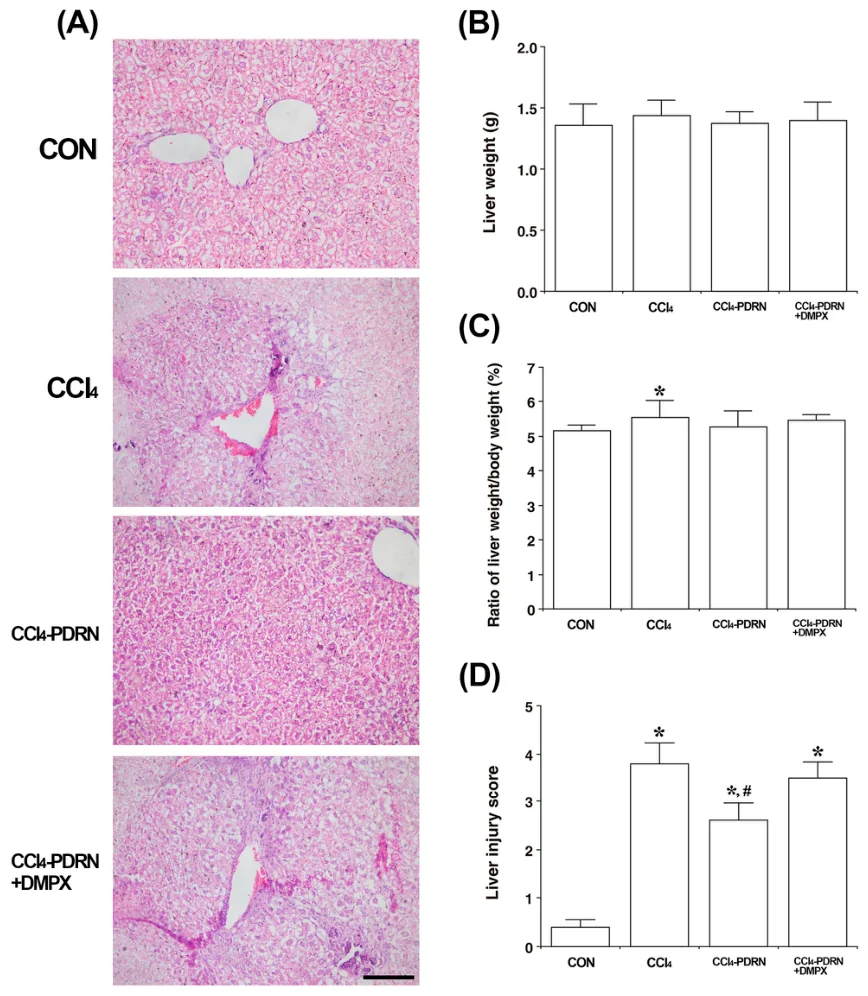
Histopathological and liver-weight findings after PDRN treatment in CCl_4_-induced ALI. (**A**) Representative H&E-stained sections of liver tissue. CCl_4_ produced hepatocyte degeneration, inflammatory infiltration, and loss of normal hepatic architecture; these pathological features were less prominent following PDRN treatment. (**B**) Absolute liver weight. (**C**) Liver-to-body weight ratio. (**D**) Histological liver injury score. Scale bar = 150 μm. (CON) Control group, (CCl_4_) CCl_4_-injected group, (CCl_4_-PDRN) CCl_4_-injected and PDRN-treated group, (CCl_4_-PDRN + DMPX) CCl_4_-injected and PDRN + DMPX-treated group. Results are shown as mean ± SEM. Sample sizes were *n* = 10/group for liver weight and liver-to-body weight ratio and *n* = 5/group for histological assessment. Differences among groups were evaluated by one-way ANOVA followed by Tukey’s HSD test. * *p* < 0.05 versus the control group; # *p* < 0.05 versus the CCl_4_-injected group.

Figure 3 presents representative H&E-stained liver sections together with the histological findings. Control liver tissue showed preserved hepatic architecture and hepatocyte morphology. In contrast, CCl_4_ administration resulted in extensive histopathological alterations including hepatocellular degeneration and inflammatory cell infiltration, together with disruption of normal hepatic structure. These changes were attenuated in mice treated with PDRN, which showed a relatively preserved hepatic architecture and reduced tissue damage. Co-administration of DMPX diminished the protective effects of PDRN, and histopathological abnormalities were more pronounced than those observed in the CCl_4_-PDRN group. Absolute liver weight did not differ significantly among the experimental groups (Figure 3B). However, the liver-to-body weight ratio was higher in the CCl_4_ group than in the control group (*p* < 0.05) (Figure 3C). Histological injury scores were also markedly elevated following CCl_4_ administration (*p* < 0.05), whereas PDRN lowered the liver injury score relative to CCl_4_ alone (*p* < 0.05). This effect was partially reversed by co-treatment with DMPX (Figure 3D).

### 3.3. PDRN Treatment Reduced Hepatic Macrophage Accumulation

**Figure 4:**
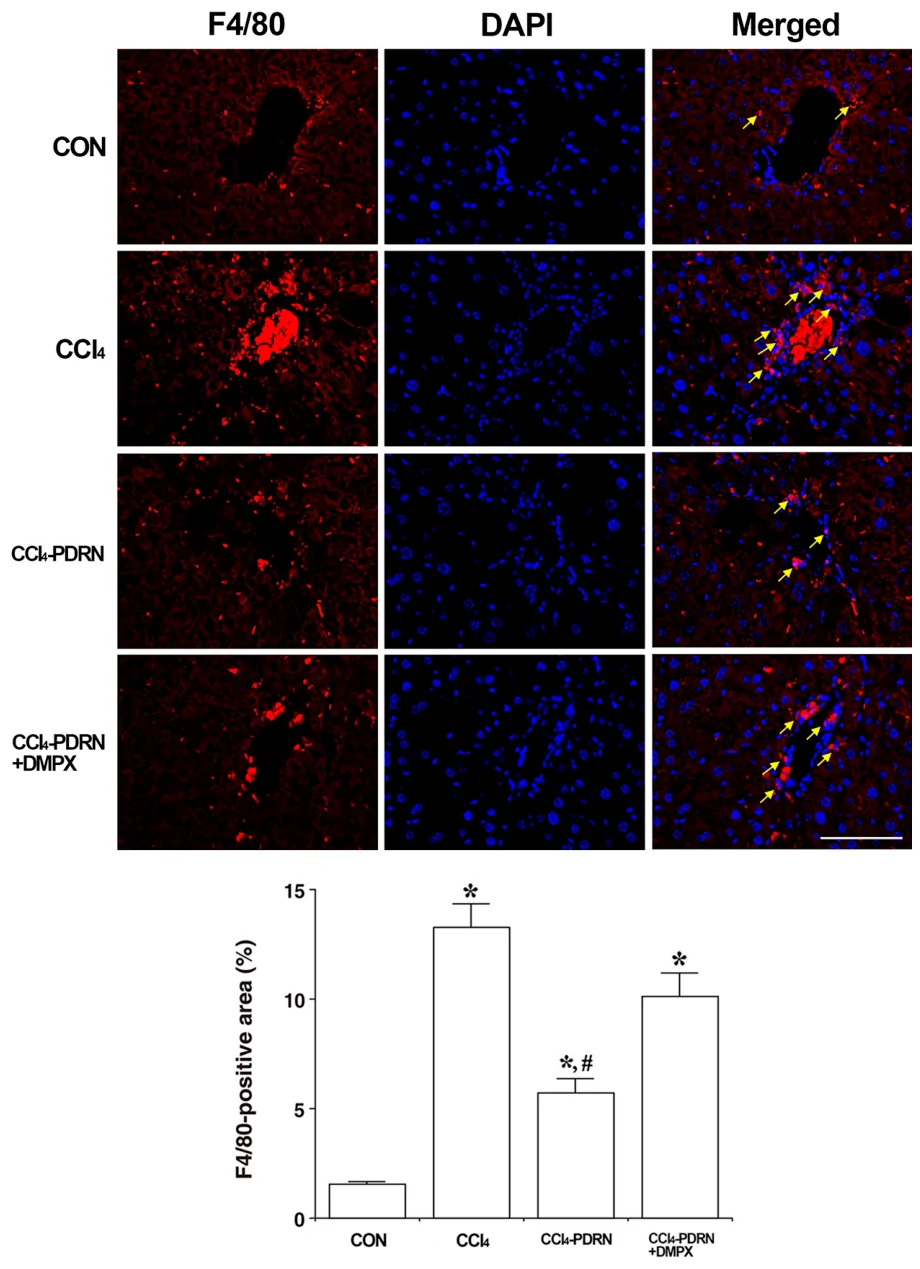
Hepatic F4/80 immunofluorescence following PDRN treatment in CCl_4_-induced ALI. Representative images show F4/80-positive macrophages (red), DAPI-labeled nuclei (blue), and merged signals across the experimental groups; arrows indicate F4/80-positive cells. Scale bar = 100 μm. The lower panel shows quantification of the F4/80-positive area. PDRN decreased the macrophage accumulation observed after CCl_4_ administration, whereas DMPX co-treatment attenuated this decrease. (CON) Control group, (CCl_4_) CCl_4_-injected group, (CCl_4_-PDRN) CCl_4_-injected and PDRN-treated group, (CCl_4_-PDRN + DMPX) CCl_4_-injected and PDRN + DMPX-treated group. Values are expressed as mean ± SEM (*n* = 5/group). Group differences were evaluated by one-way ANOVA followed by Tukey’s HSD test. * *p* < 0.05 versus the control group; # *p* < 0.05 versus the CCl_4_-injected group.

Hepatic macrophage accumulation was assessed by F4/80 immunofluorescence (Figure 4). F4/80 immunoreactivity was minimal in control liver tissue but markedly increased after CCl_4_ administration. PDRN treatment decreased the extent of F4/80 staining, whereas this decrease was diminished when DMPX was co-administered. Quantification confirmed that the F4/80-positive area was significantly greater in the CCl_4_ group than in controls (*p* < 0.05). PDRN significantly reduced the positive area, and DMPX partially attenuated this reduction (*p* < 0.05).

### 3.4. PDRN Treatment Suppressed HMGB1 and MCP-1 Expression

**Figure 5:**
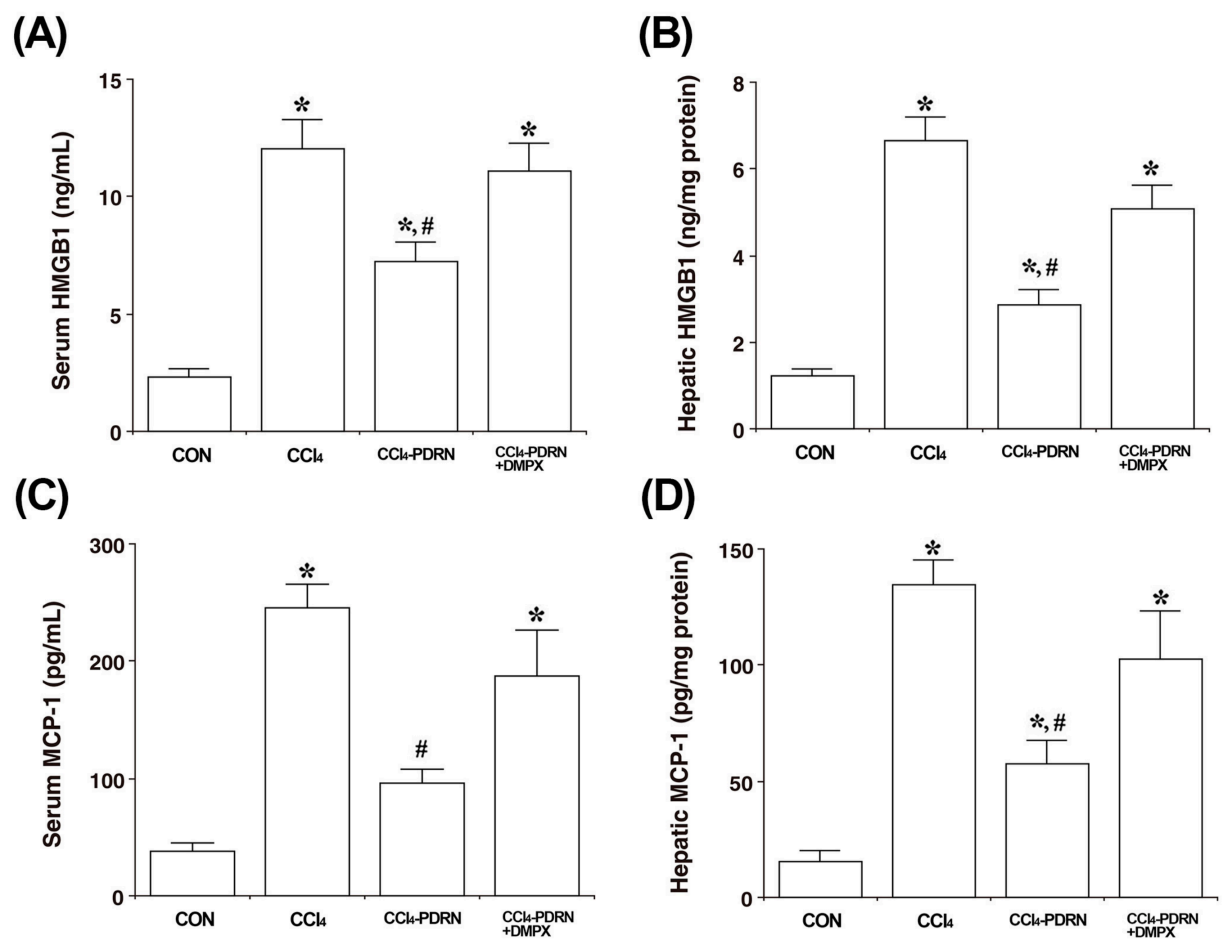
Effects of PDRN on HMGB1 and MCP-1 levels in CCl_4_-induced ALI. (**A**,**B**) Serum and hepatic HMGB1 levels, respectively; (**C**,**D**) serum and hepatic MCP-1 levels, respectively. PDRN decreased the CCl_4_-associated elevations of both mediators, whereas these reductions were attenuated by DMPX. Groups were designated as CON (control), CCl_4_ (CCl_4_-injected), CCl_4_-PDRN (CCl_4_ plus PDRN), and CCl_4_-PDRN + DMPX (CCl_4_ plus PDRN and DMPX). Values are expressed as mean ± SEM (*n* = 5/group), and group comparisons were performed by one-way ANOVA with Tukey’s HSD post hoc test. * *p* < 0.05 versus the control group; # *p* < 0.05 versus the CCl_4_-injected group.

To investigate whether PDRN modulates inflammatory mediators associated with macrophage recruitment, HMGB1 and MCP-1 levels were quantified in serum and liver tissue (Figure 5). CCl_4_ administration significantly increased serum and hepatic HMGB1 levels relative to the control group (*p* < 0.05). Treatment with PDRN significantly lowered HMGB1 in both compartments (*p* < 0.05 versus the CCl_4_ group). The co-administration of DMPX attenuated the suppressive effect of PDRN, resulting in higher HMGB1 levels than those observed in the CCl_4_-PDRN group.

A similar pattern was observed for the MCP-1 levels. Serum and hepatic MCP-1 levels were markedly elevated following CCl_4_ administration (*p* < 0.05), whereas PDRN treatment significantly decreased MCP-1 levels relative to CCl_4_ alone (*p* < 0.05). DMPX co-treatment partially reversed these effects, leading to increased MCP-1 levels in both the serum and liver tissue. Overall, reductions in HMGB1 and MCP-1 levels were accompanied by decreased hepatic macrophage accumulation following PDRN treatment.

### 3.5. PDRN Treatment Enhanced A_2A_R-Related Anti-Inflammatory Responses

**Figure 6:**
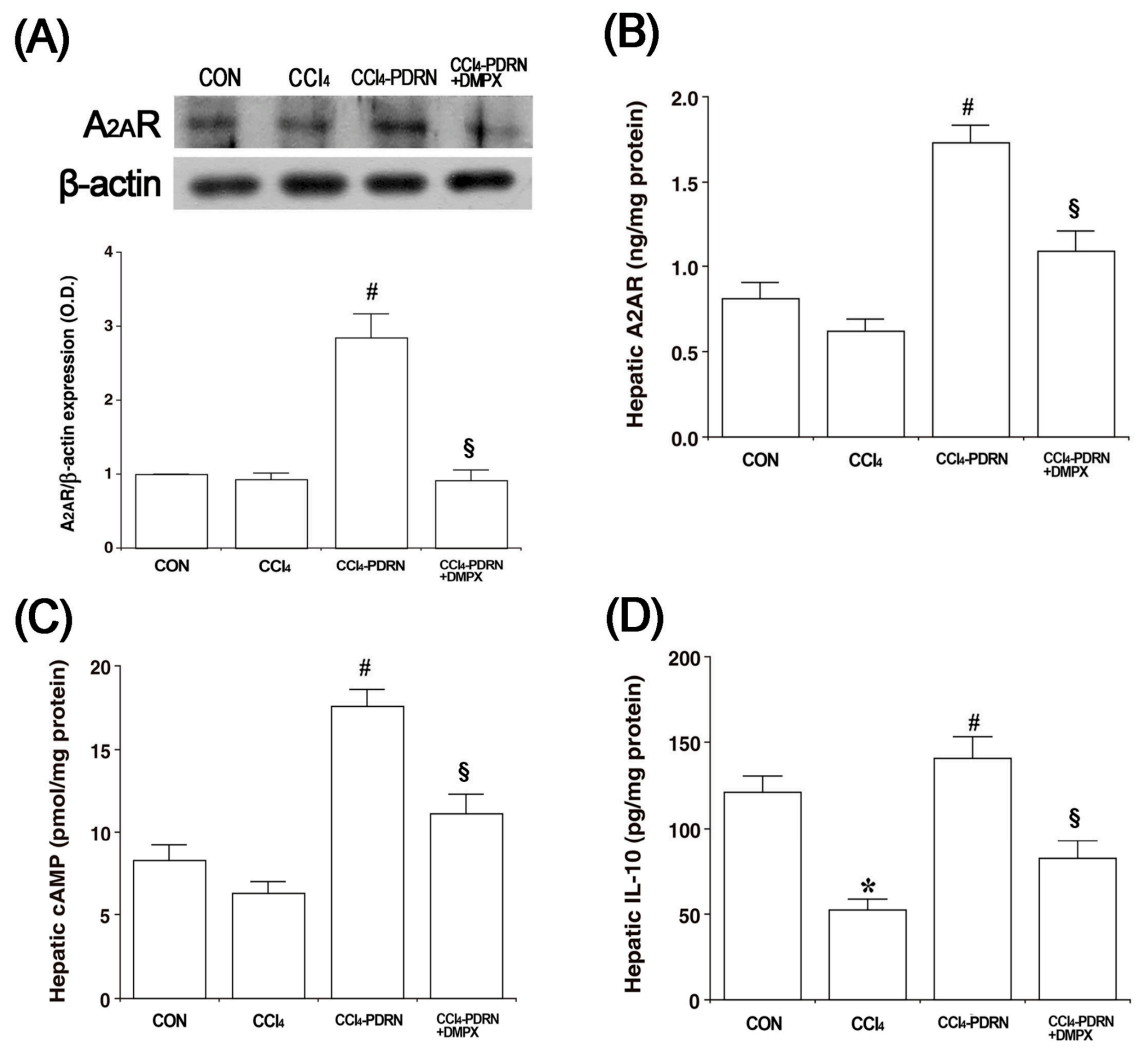
Hepatic A2AR-related responses following PDRN treatment in CCl4-induced ALI. (**A**) A2AR protein expression. (**B**) A2AR level. (**C**) cAMP level. (**D**) IL-10 level. No significant differences in these parameters were detected between the control and CCl4 groups. PDRN increased A2AR expression, cAMP, and IL-10 compared with CCl4 alone, whereas DMPX co-treatment attenuated these increases. (CON) Control group, (CCl4) CCl4-injected group, (CCl4-PDRN) CCl4-injected and PDRN-treated group, (CCl4-PDRN + DMPX) CCl4-injected and PDRN + DMPX-treated group. Data are expressed as mean ± SEM (*n* = 5/group). Differences among groups were tested by one-way ANOVA followed by Tukey’s HSD post hoc analysis. * *p* < 0.05 versus the control group; # *p* < 0.05 versus the CCl4-injected group. § *p* < 0.05 versus the CCl4-PDRN group.

Hepatic A_2A_R, cAMP, and IL-10 levels were examined to assess A_2A_R-related responses following PDRN treatment (Figure 6). A_2A_R expression tended to be lower in the CCl_4_ group than in the control group, although the difference was not statistically significant (Figure 6A). PDRN treatment significantly increased A_2A_R expression relative to the CCl_4_ group (*p* < 0.05), whereas this increase was significantly attenuated by DMPX co-treatment (*p* < 0.05). Hepatic A_2A_R and cAMP levels were significantly increased following PDRN treatment compared with the CCl_4_ group (Figure 6B,C), and these increases were significantly attenuated by DMPX co-treatment (*p* < 0.05). IL-10 levels were significantly decreased in the CCl_4_ group compared with the control group and significantly increased following PDRN treatment compared with the CCl_4_ group; this increase was significantly attenuated by DMPX co-treatment (*p* < 0.05) (Figure 6D).

## 4. Discussion

ALI is characterized by hepatocellular damage and inflammatory responses that ultimately impair liver function. Serum AST, ALT, and LDH levels are widely used biomarkers that reflect hepatocyte injury and loss of cellular integrity [22,23]. In the present study, CCl_4_ administration markedly increased serum AST, ALT, and LDH levels and induced substantial histopathological alterations in liver tissue, indicating successful establishment of ALI. These changes were significantly attenuated by PDRN treatment, suggesting that PDRN exerts protective effects against CCl_4_-induced hepatic damage. Similar hepatoprotective effects of PDRN have been previously reported, including a reduction in hepatocyte apoptosis and pro-inflammatory cytokine production through the activation of A_2A_R signaling [16,17]. These findings are consistent with previous observations demonstrating the protective effects of PDRN in experimental liver injury [16,24]. However, previous studies have primarily focused on hepatocellular injury and inflammatory signaling, whereas the relationship of PDRN treatment with HMGB1 and MCP-1 responses and hepatic macrophage accumulation during ALI has not been fully characterized. The present study extends these observations by examining these inflammatory changes together with A_2A_R-related responses.

HMGB1 has been implicated as an important mediator of hepatic inflammation and increases after hepatocellular injury [6,7]. Increased extracellular HMGB1 contributes to the amplification of inflammatory responses and the progression of liver damage [4]. In the present study, serum and hepatic HMGB1 levels were markedly elevated following CCl_4_ administration, whereas PDRN treatment significantly attenuated these increases. HMGB1 influences the expression of various inflammatory mediators involved in leukocyte recruitment and activation. Among these mediators, MCP-1 plays a central role in the recruitment of circulating monocytes and macrophages to sites of tissue injury and inflammation [11,25]. Increased MCP-1 expression has been associated with enhanced macrophage infiltration and the amplification of inflammatory responses during liver injury [9,12]. In the present study, CCl_4_ administration significantly increased the MCP-1 levels in both serum and liver tissue and was accompanied by marked accumulation of F4/80-positive macrophages within the liver. By contrast, PDRN treatment significantly attenuated MCP-1 expression and reduced hepatic macrophage accumulation. These findings suggest that the anti-inflammatory action of PDRN may be associated with reduced MCP-1 levels and hepatic macrophage accumulation during ALI. Furthermore, the concurrent reductions in HMGB1, MCP-1, and F4/80-positive macrophages following PDRN treatment suggest a close association among these inflammatory changes during ALI. These findings raise the possibility that modulation of HMGB1-associated inflammatory responses and macrophage accumulation may contribute to the hepatoprotective effects of PDRN.

The coordinated changes in HMGB1 and MCP-1 following PDRN treatment may be associated with A_2A_R-related anti-inflammatory signaling. A_2A_R activation is known to increase intracellular cAMP levels and suppress inflammatory cytokine and chemokine production in immune cells [26,27]. In addition, enhancement of A_2A_R signaling has been reported to reduce circulating MCP-1 levels and increase IL-10 production in an A_2A_R-dependent manner [28,29]. cAMP-mediated signaling also regulates macrophage responses and contributes to the resolution of inflammation [30,31]. In the present study, PDRN increased the hepatic A_2A_R, cAMP, and IL-10 levels, whereas DMPX partially reversed these effects. DMPX attenuated the inhibitory effects of PDRN on HMGB1, MCP-1, and F4/80-positive macrophage accumulation. The partial reversal of these effects by DMPX supports the involvement of A_2A_R signaling but does not indicate that the effects of PDRN are mediated exclusively through this pathway. Moreover, pharmacological antagonism with DMPX alone cannot establish definitive pathway specificity. However, the mechanism underlying the PDRN-associated increase in A_2A_R expression was not directly investigated, and additional downstream signaling pathways beyond the measured cAMP and IL-10 responses remain to be examined. These findings suggest that PDRN treatment is associated with reduced HMGB1 and MCP-1 expression, decreased hepatic macrophage accumulation, and enhanced A_2A_R-related responses.

**Figure 7:**
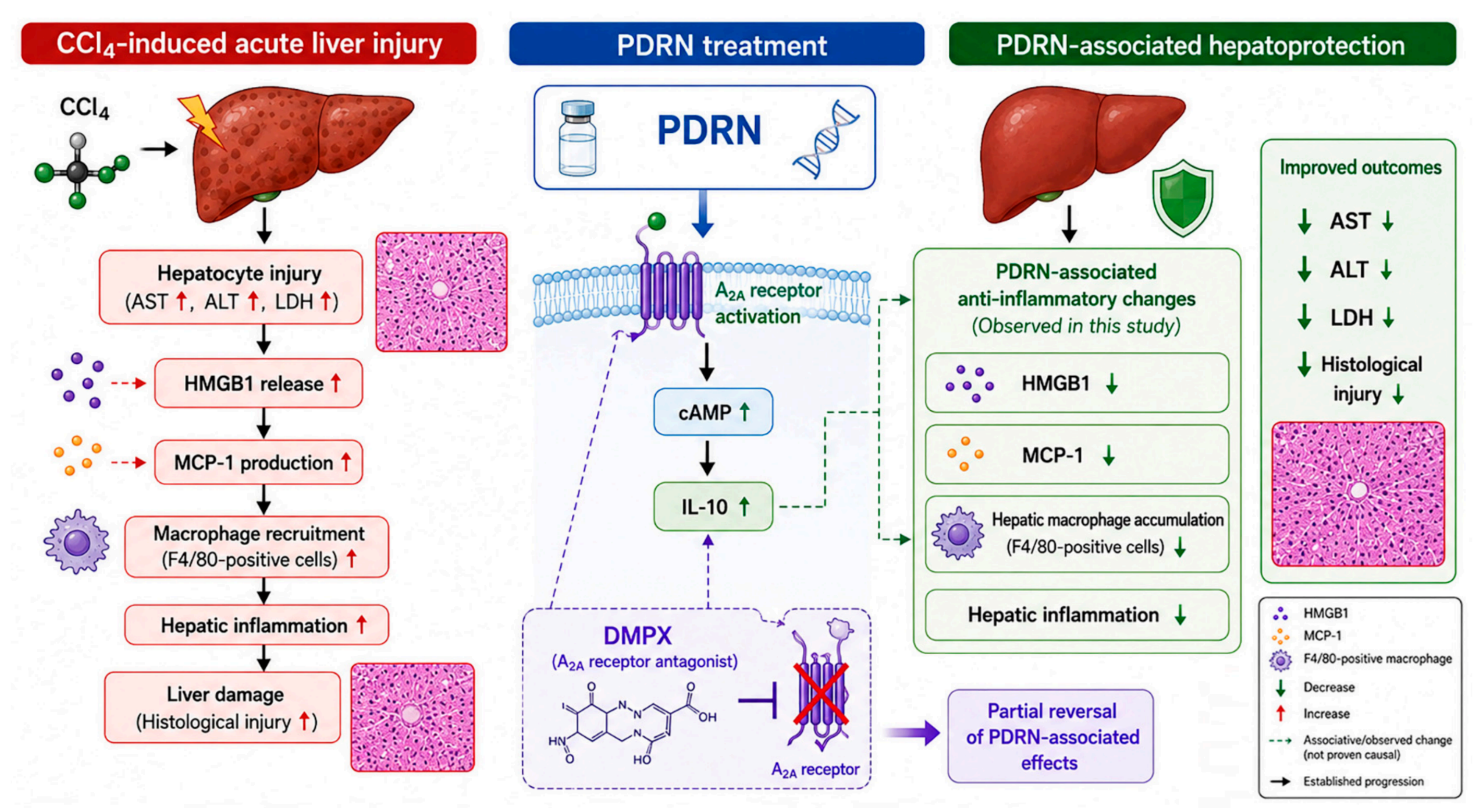
Schematic representation of the main findings of the present study. PDRN enhanced A_2A_R-related responses and was associated with reductions in HMGB1 and MCP-1 levels, hepatic macrophage accumulation, and liver injury following CCl_4_ administration. DMPX partially reversed these effects.

Although the present study demonstrated coordinated changes in HMGB1, MCP-1, and hepatic macrophage accumulation following PDRN treatment, the causal relationships among these events were not directly investigated. Targeted modulation of HMGB1 or MCP-1 will therefore be required to establish their causal involvement. In addition, key pro-inflammatory signaling pathways were not directly examined in the present study, limiting mechanistic interpretation of the observed anti-inflammatory response. F4/80 staining also does not distinguish macrophage subpopulations, limiting further interpretation of macrophage-specific responses. In addition, the present findings should be interpreted within the context of an acute CCl_4_-induced liver injury model, and their applicability to other forms of liver injury remains to be determined. Further studies are needed to clarify the precise mechanisms underlying the hepatoprotective effects of PDRN. Despite these limitations, the present findings provide evidence of coordinated anti-inflammatory changes associated with PDRN treatment during ALI. While previous studies have demonstrated the hepatoprotective effects of PDRN in experimental liver injury, the present study extends these observations by demonstrating coordinated changes in HMGB1 and MCP-1 levels, hepatic macrophage accumulation, and A_2A_R-related responses following PDRN treatment. Although these findings do not establish a direct HMGB1-MCP-1 signaling axis, they provide additional evidence linking the anti-inflammatory effects of PDRN to these inflammatory responses during ALI. The main findings of the present study are summarized schematically in Figure 7.

## 5. Conclusions

PDRN attenuated CCl_4_-induced ALI and reduced HMGB1 and MCP-1 levels as well as hepatic macrophage accumulation. These changes were accompanied by increased A_2A_R and cAMP levels and were partially reversed by DMPX administration. Collectively, the present findings suggest that modulation of HMGB1-associated inflammatory responses and hepatic macrophage accumulation may contribute to the hepatoprotective effects of PDRN in ALI. As these findings were obtained using a CCl_4_-induced ALI model, their applicability to other forms of liver injury remains to be established. These findings provide additional insight into the anti-inflammatory responses associated with PDRN treatment and support further investigation of its therapeutic potential for ALI.

## Data Availability

Data supporting the findings of this study are included in the manuscript, and additional experimental information can be provided upon reasonable request.

## Abbreviations

Abbreviations used throughout the manuscript are listed below:

ALI	Acute liver injury
PDRN	Polydeoxyribonucleotide
A_2A_R	Adenosine A_2A_ receptor
HMGB1	High mobility group box 1
DMPX	3,7-dimethyl-1-propargylxanthine
MCP-1	Monocyte chemoattractant protein-1
CCl4	Carbon tetrachloride
IL-10	Interleukin-10
H&E	Hematoxylin and eosin
cAMP	cyclic adenosine monophosphate
ELISA	Enzyme-linked immunosorbent assay
AST	Aspartate aminotransferase
ALT	Alanine aminotransferase
LDH	Lactate dehydrogenase
